# Elucidating the Neuroprotective Effect of *Tecoma stans* Leaf Extract in STZ-Induced Diabetic Neuropathy

**DOI:** 10.1155/2022/3833392

**Published:** 2022-06-26

**Authors:** Amit Gupta, Tapan Behl, Aayush Sehgal, Sukhbir Singh, Neelam Sharma, Shivam Yadav, Khalid Anwer, Celia Vargas-De-La Cruz, Sridevi Chigurupati, Abdullah Farasani, Saurabh Bhatia

**Affiliations:** ^1^Chitkara College of Pharmacy, Chitkara University, Rajpura, Punjab, India; ^2^Yashraj Institute of Pharmacy, Lucknow, Uttar Pradesh, India; ^3^Department of Pharmaceutics, College of Pharmacy, Prince Sattam Bin Abdulaziz University, Alkharj, Saudi Arabia; ^4^Department of Pharmacology, Bromatology and Toxicology, Faculty of Pharmacy and Biochemistry, Universidad Nacional Mayor de San Marcos, Lima, Peru; ^5^E-Health Research Center, Universidad de Ciencias y Humanidades, Lima, Peru; ^6^Department of Medicinal Chemistry and Pharmacognosy, College of Pharmacy, Qassim University, Buraidah 52571, Saudi Arabia; ^7^Department of Biotechnology, Saveetha College of Engineering, Saveetha Institute of Medical and Technical sciences, Saveetha University, Saveetha Nagar, Thandalam, Chennai 602105, India; ^8^Biomedical Research Unit, Medical Research Center, Jazan University, Jazan, Saudi Arabia; ^9^Department of Medical Laboratory Technology, College of Applied Medical Sciences, Jazan University, Jazan, Saudi Arabia; ^10^Natural & Medical Sciences Research Centre, University of Nizwa, Birkat Al Mauz, Nizwa, Oman; ^11^School of Health Science, University of Petroleum and Energy Studies, Dehradun, Uttarakhand, India

## Abstract

**Background:**

Diabetes is considered one of the most encyclopedic metabolic disorders owing to an alarming rise in the number of patients, which is increasing at an exponential rate. With the current therapeutics, which only aims to provide symptomatic and momentary relief, the scientists are shifting gears to explore alternative therapies which not only can target diabetes but can also help in limiting the progression of diabetic complications including diabetic neuropathy (DN).

**Methods:**

*Tecoma stans* leaf methanolic extract was prepared using the Soxhlet method. A streptozotocin (STZ; 45 mg/kg)-induced diabetic animal model was used and treatment with oral dosing of *T. stans* leaf extract at the different doses of 200 mg/kg, 300 mg/kg, and highest dose, i.e., 400 mg/kg, was initiated on day 3 after STZ administration. The pharmacological response for general and biochemical (angiogenic, inflammatory, and oxidative) parameters and behavioral parameters were compared using Gabapentin as a standard drug with the results from the test drug.

**Results:**

Parameters associated with the pathogenesis of diabetic neuropathy were evaluated. For general parameters, different doses of *T. stans* extract (TSE) on blood sugar showed significant effects as compared to the diabetic group. Also, the results from biochemical analysis and behavioral parameters showed significant positive effects in line with general parameters. The combination therapy of TSE at 400 mg/kg with a standard drug produced nonsignificant effects in comparison with the normal group.

**Conclusion:**

The leaves of *T. stans* possess antidiabetic effects along with promising effects in the management of DN by producing significant effects by exhibiting antioxidative, antiangiogenic, and anti-inflammatory properties, which are prognostic markers for DN, and thus, *T. stans* can be considered as an emerging therapeutic option for DN.

## 1. Introduction

Diabetes mellitus is nowadays considered one of the “lifestyle” disorders, and over centuries, various species of plants are considered a fundamental resource of potent hypoglycaemic agents [1]. In developing nations, specifically, medicinal agents are utilized to treat DM to overcome the cost burden of conventional medications on the population [2]. Since decades, plants and their byproducts have been used as a major source of medicines due to their therapeutic potential. In recent times, treating diseases including DM utilizing medicinal plant agents is suggested as these plants possess numerous phytoconstituents, namely, glycosides, alkaloids, saponins, flavonoids, carotenoids, and terpenoids, which show hypoglycaemic activity [3]. In one of the recent studies, it was postulated that the synergistic effect of biologically active constituents such as glucosinolates, lignans, polyphenols, coumarins, and carotenoids leads to the major beneficial characteristics of every plant matrix and this signifies the initial step for understanding its beneficial actions and biological activities [4]. The hypoglycaemic actions which result after treatment with plant extracts are generally ascribed to their capability to increase the pancreatic gland tissue performance, which is achieved by decreasing the intestinal absorption of sugar or by enhancing insulin hormone secretions [5].

There is an array of literature available, which describes the potential of natural products derived from plant origin, which have the potential to become the drug of choice in various metabolic disorders. These products, when used as monotherapy or in combination with current allopathic drugs, were shown to decrease the therapeutic dose, however achieving the same pharmacological effects [6]. The least adverse effects, low cost, and easy availability make these plant-based formulations the major option among all the available treatments, specifically in rural regions [7]. On the other hand, numerous plants are rich resources of bioactive agents that are free from detrimental adverse effects and exhibit excellent pharmacological actions [8, 9].

Various studies carried out over the past few decades confirm the therapeutic potential of various herbal plants in the management of diabetes and diabetes-associated complications [10]. From these plants, promising results are also obtained from *T. stans*, which is considered a drug of choice in Mexico for the management of diabetes. From the available evidence in the published literature, the tremendous potential of *T. stans* in the management of various disorders can be easily deduced due to the presence of an array of active phytoconstituents. This was evident from the literature that *T. stans* possesses multiple pharmacological properties which include anticancer, cardioprotective, wound-healing, anti-inflammatory, neuroprotective, antimicrobial, and various other pathological properties, which are involved directly and indirectly in the pathogenesis of various metabolic disorders [10]. Multiple studies in the past had revealed several other uses of different extracts of *T. stans* apart from its antidiabetic properties. Studies till date reported anticancer activity in vivo and in vitro of methanolic extracts of *T. stans* flowers [11], antinociceptive and anti-inflammatory potential of flower extract *T. stans* [12], antioxidant and cytotoxic activity of *T. stans* leaves [13] along with antidiabetic effects on STZ induced diabetic rats.

An array of in vitro and in vivo studies had been conducted in the past few decades, which had confirmed the tremendous antidiabetic potential of *T. stans*. The available literature and studies confirm that the plant leaves, flowers, hardwood, and whole plant can be used to procure active pharmaceutical ingredients, which can elicit therapeutic properties. The antidiabetic effects were mainly mediated by the presence of alkaloids, flavonoids, and glycosides, which have been shown to decrease fasting glycemic levels, an area under the glucose tolerance curve [14]. These active constituents can also decrease plasma cholesterol levels and can exhibit antifungal effects, antimicrobial effects, and even hepatoprotective effects [15]. In addition, the methanolic extract of *T. stans* is known to possess activities that includes intestinal *α*-glucosidase inhibition, decreasing hypoglycaemic peaks, and even affecting postprandial antidiabetic effects [16]. These effects are not only limited to the management of diabetes but can also help in reducing triglyceride levels including cholesterol without any changes in blood fasting glucose levels and is also associated with the management of various pathological conditions [17].

It has been already confirmed from various research that *T. stans* leaf extract contains the monoterpenoid alkaloids such as tecostanine and tecomine, saponins, and flavonoids, which have a hypoglycaemic effect and exert varied effects in multiple pathological conditions. However, no study has been carried out and is available in the literature to explore the effect of *T. stans* in diabetic neuropathy. Hence, through the undertaken study, we tried to explore the effect of *T. stans* on diabetic neuropathy, which is the most common complication associated with diabetes [18].

## 2. Materials and Methods

### 2.1. Plant Material

Fresh leaves of *T. stans* were collected from Jaswant Green Nursery, Punjab, India. The leaves were authenticated by the National Institute of Pharmaceutical Education and Research (NIPER), Mohali, Punjab, with reference number NIP-H-286. The voucher specimen was deposited and was stored in a publicly available herbarium of the institute.

### 2.2. Extract Preparation

The leaves were collected (5.0 kg), washed thoroughly with tap water, and dried. They were coarsely powdered (4–6 mm) with simultaneous removal of ground material (1.3 kg) with methanol solution (60%, 1 : 10 ratio, w/v) at 60°C for 30 min. This was followed by their extraction with a Soxhlet apparatus using 60 g of dried coarse powder with 300 ml of methanol for 6–8 h at room temperature to prepare the methanolic extract. The remaining drug was dried to remove the methanol followed by maceration of the dried powder with distilled water for 48 h. Later, marc was pressed, and the extract was collected. The volume was further concentrated using a rotary vacuum evaporator at a temperature not exceeding 60°C for 30–40 minutes at 500 mm of Hg until it formed a paste which was further dried till it was converted to powder and stored at 40°C in a well-closed container for further analysis [19].

### 2.3. Chemical Used

All the chemicals used in the study were collected from the store of Chitkara College of Pharmacy, Chitkara University, and of analytical grade manufactured by Loba Chemie. The standard drug, Gabapentin, was a benevolent gift sample from Magbro Healthcare Pvt. Ltd., Solan, India. The biochemical kits were obtained from allied scientific products.

### 2.4. Animals

This study involves the in vivo evaluation of *T. stans* in Wistar rats (200–250 gm), irrespective of their sex. These animals were approved by Institutional Animal Ethic Committee (IAEC) from Chitkara College of Pharmacy, Chitkara University, Punjab. The animals were kept in controlled conditions as per the standard procedures having adequate humidity and ventilation with proper lighting. The room temperature was maintained at 23 ± 1°C. The care of these animals was undertaken as per the guidance obtained from the committee for the purpose of control and supervision of experimental animals (CDCSEA), and the research protocol was submitted to the IAEC under the protocol no. IAEC/CCP/21/02/PR-007.

Diabetes in these animals was induced using STZ with a single administration of intraperitoneal (i.p.) streptozotocin (STZ) injection (dose of 45 mg/kg) in 0.1 M citrate buffer having a pH value of 4.5 in overnight fasted Wistar rats [20]. The preparation of citrate buffer having a pH value of 4.5 was carried out by adding 25.5 ml of 0.1 M citric acid solution (19.2 g/1000 ml distilled water) and 24.5 ml of 0.1 M sodium citrate dehydrate (C_6_H_9_Na_3_O_9_) in 29.4 g/1000 ml of distilled water, and after that, pH was confirmed using a pH meter [21]. Blood glucose levels were estimated prior to induction of diabetes and 48 hours after STZ injection. Blood glucose levels in all experimental groups were estimated with a glucometer along with glucose test strips. Animals with more than 300 mg/dl blood glucose values were considered diseased (diabetic control) and were considered part of this present study [22]. The experimental animals were randomly assigned to the following groups and were marked accordingly:

- Group 1: normal control
- Group 2: diabetic control
- Group 3: *T. stans* per se
- Group 4: Gabapentin (50 mg/kg body weight)-treated group
- Group 5: *T. stans* extract 200 mg/kg-treated group
- Group 6: *T. stans* extract 300 mg/kg-treated group
- Group 7: *T. stans* extract 400 mg/kg-treated group
- Group 8: *T. stans* extract 400 mg/kg + Gabapentin-treated group

All these groups were analyzed for confirmation of STZ-induced diabetes by general parameter estimation of blood glucose levels, biweekly, and increased level of blood glucose and subsequent mortality were controlled by administration of huminsulin (1 unit of huminsulin if blood glucose is greater than 500 mg/dl and 2 units if value was greater than 600 mg/dl). Also, the biochemical parameters for the confirmation of angiogenesis, oxidation, and inflammation were analyzed using ELISA kits. These tests were further backed with the behavioral parameters, which were further analyzed to monitor the effect of test and standard drugs in STZ-induced diabetic neuropathy and associated secondary complications.

### 2.5. Statistical Analysis

For statistical analysis, one-way and two-way ANOVA using GraphPad PRISM version 9.0 was used and the interpretation was carried out as mean ± SD (*n* = 6). The results involved the post hoc use of Tukey–Kramer's multiple comparisons. The level of statistical significance was expressed at *p* < 0.05, *p* < 0.01, and *p* < 0.001.

## 3. Results

The dried coarse powdered leaves were subjected to continuous hot extraction by using methanol as the solvent. From extraction of leaves of *T. stans* by the continuous hot percolation method using methanol, the average yield was 11.6% w/w. Furthermore, 3 different doses were prepared to determine the activity of methanolic extract of *T. stans* leaves at different doses of 200 mg/kg, 300 mg/kg, and 400 mg/kg. Its activity was determined by analyzing general parameters like changes in blood glucose levels. These results from the test drugs were then compared with those using a standard drug, which included Gabapentin. From the literature evidence, it can be concluded that the standard drug exhibits a statistically significant effect in maintaining these physical parameters in comparison with the disease control group [23–25]; however, the effects of Gabapentin in regulating the blood glucose is limited. The experimental rats administered with *T. stans* extract also showed statistically significant results in terms of effectiveness and improvements against the disease control group. Also, using the highest dose in combination with the standard drugs either produced additive effects, or nonsignificant results were seen in comparison to the normal rats.

### 3.1. General Parameters

The study includes an estimation of general parameters, which are required for the confirmation of diabetes in the present study. A general confirmatory test, which includes blood glucose, was done, which confirmed that diabetes was induced in the experimental Wistar rats, which were further evaluated for diabetes-related complications of neuropathy. Diabetic neuropathy was further evaluated using biochemical and behavioral parameters.

#### 3.1.1. Estimation of Blood Glucose Levels

**Figure 1:**
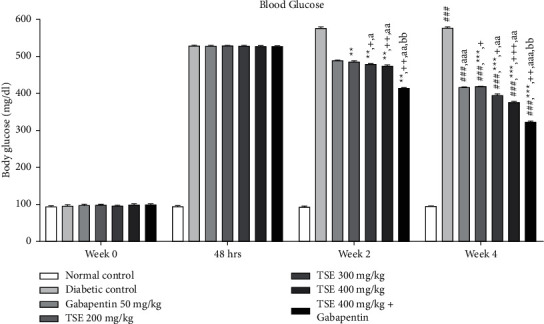
Effect of *T. stans* extract on blood glucose levels. Data are expressed as mean ± SD (*n* = 6). Data were statistically analyzed using two-way ANOVA followed by Tukey–Kramer's multiple comparison test at *p* < 0.05, *p* < 0.01, and *p* < 0.001. The values were expressed as ^#^*p* < 0.05, ^##^*p* < 0.01, and ^###^*p* < 0.001 vs. normal control group at the 4th week; *p*^*∗*^ < 0.05, ^*∗∗*^*p* < 0.01, and ^*∗∗∗*^*p* < 0.001 vs. diabetic control group; ^+^*p* < 0.05, ^++^*p* < 0.01, and ^+++^*p* < 0.001 vs. standard treatment with Gabapentin; ^*a*^*p* < 0.05 and ^*aa*^*p* < 0.01 vs. TSE 200 mg/kg; and ^*b*^*p* < 0.05 and ^*bb*^*p* < 0.01 vs. TSE 300 mg/kg.

The activity of methanolic extract of *T. stans* leaves at different doses of 200 mg/kg, 300 mg/kg, and 400 mg/kg was determined by analyzing general parameters like increase/decrease in blood glucose levels. These results from the test drugs were then compared to those using a standard drug, which included Gabapentin. The results from this experiment concluded that experimental rats administered with *T. stans* extract also showed statistically significant results in terms of effectiveness and improvements against the disease control group but were not able to achieve normal glycemic levels as compared to normal rats as shown in Figure 1. However, using the highest dose, i.e., 400 mg/kg, in combination with the standard drug produced nonsignificant results that were seen in comparison to the standard treatment alone. This confirms the dose saturation observed at the dose of 400 mg/kg when used as a combination therapy.

### 3.2. Determination of Angiogenic Parameters

Angiogenesis is purported as de novo synthesis of new blood vessels. From the published literature, angiogenesis has been considered one of the primary triggers, which is involved in the pathogenesis of diabetes-related complications of neuropathy [26]. The main factors responsible for the pathogenesis resulting in abnormal/excessive formation of new blood vessels are mediated by downstream mediators, vascular endothelial growth factor (VEGF) and protein kinase C (PKC), which were augmented in the serum postadministration of STZ [27]. Diabetic rats administered with *T. stans* extract of 200 mg/kg, 300 mg/kg, and the highest dose, i.e., 400 mg/kg, along with the standard treatment Gabapentin showed a significant decrease in the levels of angiogenic markers to the normalization levels in a dose-dependent manner.

#### 3.2.1. Estimation of PKC-*β* Levels

**Figure 2:**
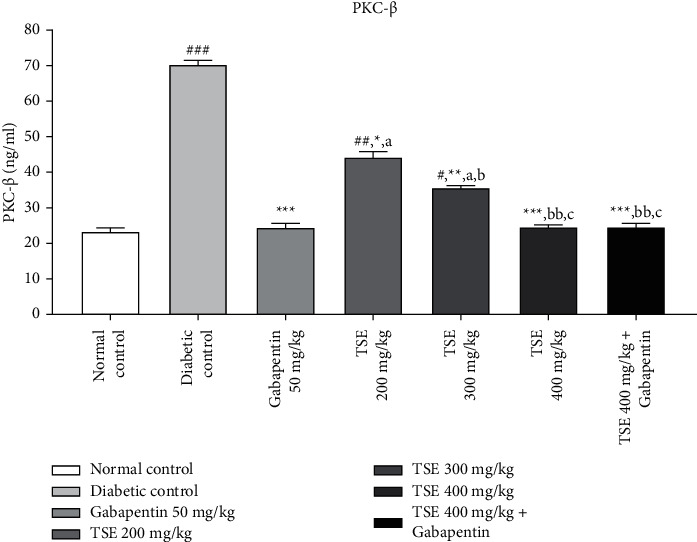
Estimation of PKC-*β* levels after completion of the study protocol. Data are expressed as mean ± SD (*n* = 6). Data were statistically analyzed using one-way ANOVA followed by Tukey–Kramer's multiple comparison test at *p* < 0.05, *p* < 0.01, and *p* < 0.001. The values are expressed as ^#^*p* < 0.05, ^##^*p* < 0.01, and ^###^*p* < 0.001 compared with the normal control group; ^*∗*^*p* < 0.05, ^*∗∗*^*p* < 0.01, and ^*∗∗∗*^*p* < 0.001 compared with the diabetic control group; ^*a*^*p* < 0.05 compared with standard treatment Gabapentin; ^*b*^*p* < 0.05 and ^*bb*^*p* < 0.01 compared with TSE 200 mg/kg; and ^*c*^*p* < 0.05 compared with TSE 300 mg/kg.

From the published literature, it is evident that protein kinase C (PKC) is considered a crucial marker in determining angiogenesis. Postadministration of STZ in Wistar rats results in increased levels of PKC, which is involved in the pathogenesis of diabetes and associated complications [28]. Administration of *T. stans* extracts at 200 mg/kg, 300 mg/kg, and the highest dose, i.e., 400 mg/kg, along with the standard treatment Gabapentin showed a significant decrease in the levels of PKC in comparison to the disease control group. In addition, in the combination treatment of the highest dose of *T. stans*, i.e., 400 mg/kg with the standard drug, Gabapentin, produced nonsignificant differences in PKC-*β* levels in maintaining the level of PKC-*β* to the levels found in normal rats as shown in Figure 2.

#### 3.2.2. Estimation of VEGF Levels

**Figure 3:**
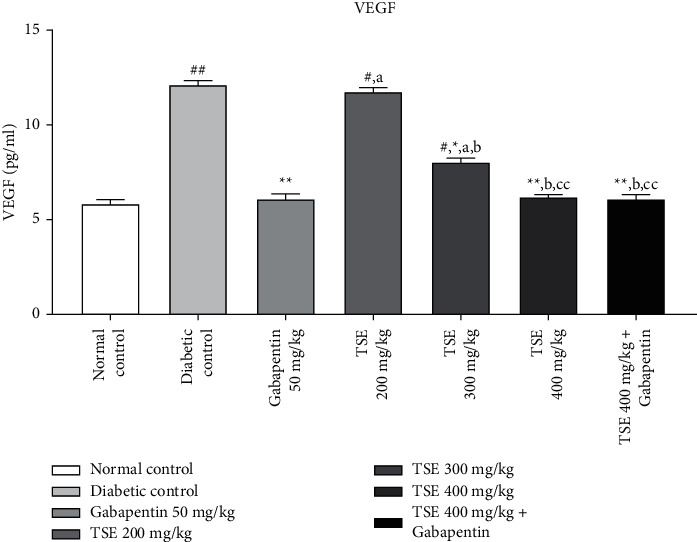
Estimation of VEGF levels after completion of the study protocol. Data are expressed as mean ± SD (*n* = 6). Data were statistically analyzed using one-way ANOVA followed by Tukey–Kramer's multiple comparison test at *p* < 0.05, *p* < 0.01, and *p* < 0.001. The values are expressed as ^#^*p* < 0.05 and ^##^*p* < 0.01 compared with the normal control group; ^*∗*^*p* < 0.05 and ^*∗∗*^*p* < 0.01 compared with the diabetic control group; ^*a*^*p* < 0.05 compared with standard treatment Gabapentin; ^*b*^*p* < 0.01 compared with TSE 200 mg/kg; and ^*c*^*p* < 0.05 and ^*cc*^*p* < 0.01 compared with TSE 300 mg/kg.

VEGF is a potent biomarker and is considered a parameter for the determination of angiogenesis. From the available literature, it can be deduced that an increase in levels of VEGF is associated in the animal model of diabetes induced by the administration of STZ [29]. Administration of *T. stans* extract at 200 mg/kg, 300 mg/kg, and the highest dose, i.e., 400 mg/kg, along with the standard treatment Gabapentin showed a significant decrease in the levels of VEGF in comparison to the disease control group [30]. In addition, the combination treatment of the highest dose of *T. stans*, i.e., 400 mg/kg with the standard drug, Gabapentin, produced nonsignificant differences in maintaining the level of VEGF to the levels found in normal rats as shown in Figure 3.

### 3.3. Determination of Inflammatory Parameters

The levels of tumor necrosis factor alpha (TNF-*α*) and interleukin 1 beta (IL-1*β*) were augmented in the serum of rats after administration of STZ. Diabetic Wistar rats treated with *T. stans* extract at different doses of 200 mg/kg, 300 mg/kg, and the highest dose, i.e., 400 mg/kg along with the standard treatment Gabapentin significantly drained the levels of inflammatory mediators such as IL-1*β* and TNF-*α* to the levels found in the normal rats.

#### 3.3.1. Estimation of IL-*β* Levels

**Figure 4:**
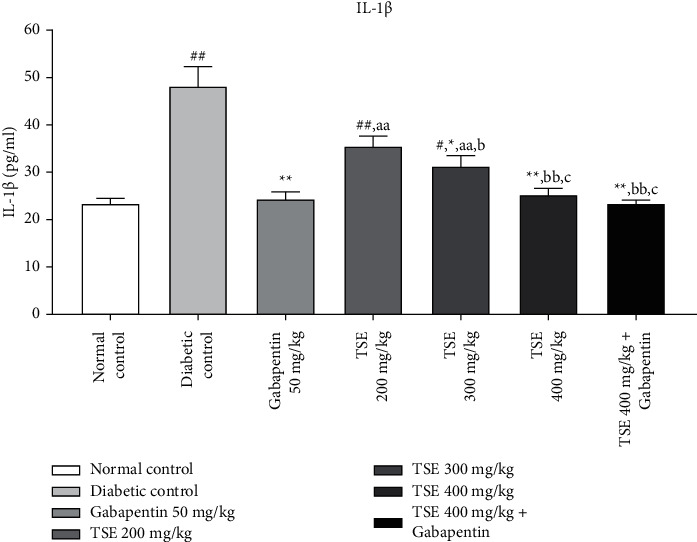
Estimation of IL-*β* levels after completion of the study protocol. Data are expressed as mean ± SD (*n* = 6). Data were statistically analyzed using one-way ANOVA followed by Tukey–Kramer's multiple comparison test at *p* < 0.05, *p* < 0.01, and *p* < 0.001. The values are expressed as ^#^*p* < 0.05 and ^##^*p* < 0.01 compared with the normal control group; ^*∗*^*p* < 0.05 and ^*∗∗*^*p* < 0.01 compared with the diabetic control group; ^*a*^*p* < 0.05 and ^*aa*^*p* < 0.01 compared with standard treatment, Gabapentin; ^*b*^*p* < 0.05 and ^*bb*^*p* < 0.01 compared with TSE 200 mg/kg; and ^*c*^*p* < 0.05 compared with TSE 300 mg/kg.

The levels of proinflammatory cytokines (IL-1*β*) are exceptionally high in conditions of diabetes-associated complications [31, 32]. Administration of *T. stans* extracts at 200 mg/kg, 300 mg/kg, and the highest dose, i.e., 400 mg/kg, along with the standard treatment Gabapentin showed a significant decrease in the levels of VEGF in comparison to the disease control group. In addition, in the combination treatment of the highest dose of *T. stans*, i.e., 400 mg/kg with the standard drug, Gabapentin, produced nonsignificant differences in maintaining the level of IL-1*β* to the levels found in normal rats as shown in Figure 4.

#### 3.3.2. Estimation of TNF-*α* Levels

**Figure 5:**
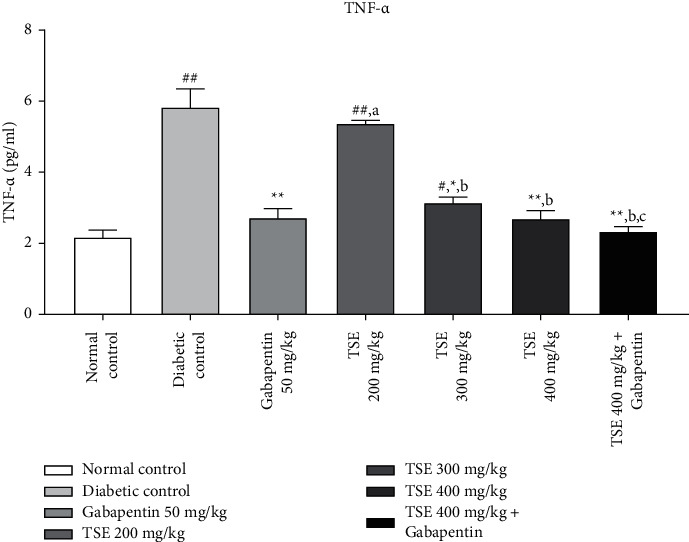
Estimation of TNF-*α* levels after completion of the study protocol.

TNF-*α* is a common biomarker that depicts the extent of inflammation in any tissue. From the literature evidence, it has been found that the levels of TNF-*α* significantly increase in an animal model of diabetes administered with STZ. In this study, administration of *T. stans* extracts at 200 mg/kg, 300 mg/kg, and the highest dose, i.e., 400 mg/kg, along with the standard treatment Gabapentin showed a significant decrease in the levels of TNF-*α* in comparison to the disease control group [33]. In addition, the combination treatment of the highest dose of *T. stans* extract, i.e., 400 mg/kg, with the standard drug, Gabapentin, produced nonsignificant differences in maintaining the level of TNF-*α* to the levels found in normal rats as shown in Figure 5.

Data are expressed as mean ± SD (*n* = 6). Data were statistically analyzed using one-way ANOVA followed by Tukey–Kramer's multiple comparison test at *p* < 0.05, *p* < 0.01, and *p* < 0.001. The values are expressed as ^#^*p* < 0.05 and ^##^*p* < 0.01 compared with the normal control group; ^*∗*^*p* < 0.05 and ^*∗∗*^*p* < 0.01 compared with the diabetic control group; ^*a*^*p* < 0.05 compared with standard treatment Gabapentin; ^*b*^*p* < 0.05 compared with TSE 200 mg/kg; and ^*c*^*p* < 0.05 compared with TSE 300 mg/kg.

### 3.4. Determination of Oxidation Paramaters

In this study, the antioxidant parameters were also measured using ELISA kits. From the literature evidence, it was confirmed that an increase in the activity of the malondialdehyde (MDA) enzyme was predominant in the diabetic animal models after treatment of STZ injection with a simultaneous decrease in catalase and superoxide dismutase (SOD) activity [34, 35]. In this study, administration of *T. stans* extract at 200 mg/kg, 300 mg/kg, and 400 mg/kg and standard treatment Gabapentin significantly altered the antioxidant enzymes to the levels found in the normal rats.

#### 3.4.1. Estimation of Catalase Levels

**Figure 6:**
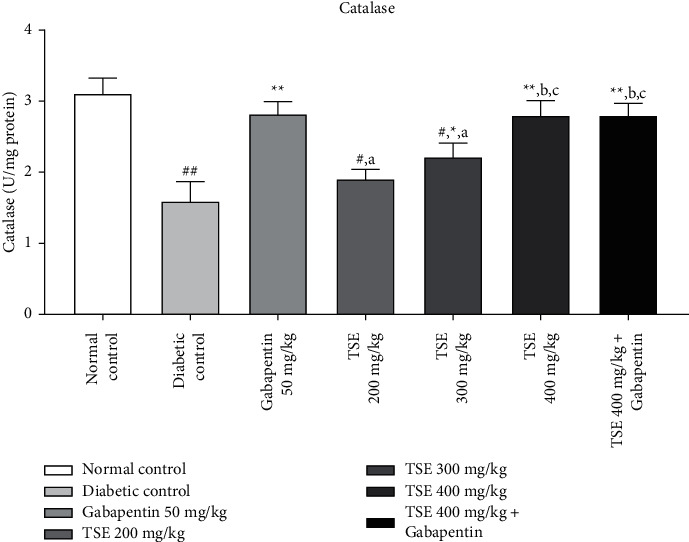
Estimation of catalase level after completion of the study protocol. Data are expressed as mean ± SD (*n* = 6). Data were statistically analyzed using one-way ANOVA followed by Tukey–Kramer's multiple comparison test at *p* < 0.05, *p* < 0.01, and *p* < 0.001. The values are expressed as ^#^*p* < 0.05 and ^##^*p* < 0.01 compared with the normal control group; ^*∗*^*p* < 0.05 and ^*∗∗*^*p* < 0.01 compared with the diabetic control group; ^*a*^*p* < 0.05 compared with standard treatment, Gabapentin; ^*b*^*p* < 0.05 compared with TSE 200 mg/kg; and ^*c*^*p* < 0.05 compared with TSE 300 mg/kg.

From the published literature, it is evident that the level of catalase dropped extensively in the disease control animal group and is responsible for decreasing antioxidant activity. Administration of *T. stans* extract at different doses of 200 mg/kg, 300 mg/kg, and the highest dose, i.e., 400 mg/kg, along with the standard treatment Gabapentin in disease control animals showed a statistically significant escalation in the levels of catalase in comparison against disease control [36]. The combination produced statistically nonsignificant effects vs. Gabapentin as shown in Figure 6.

#### 3.4.2. Estimation of SOD Levels

**Figure 7:**
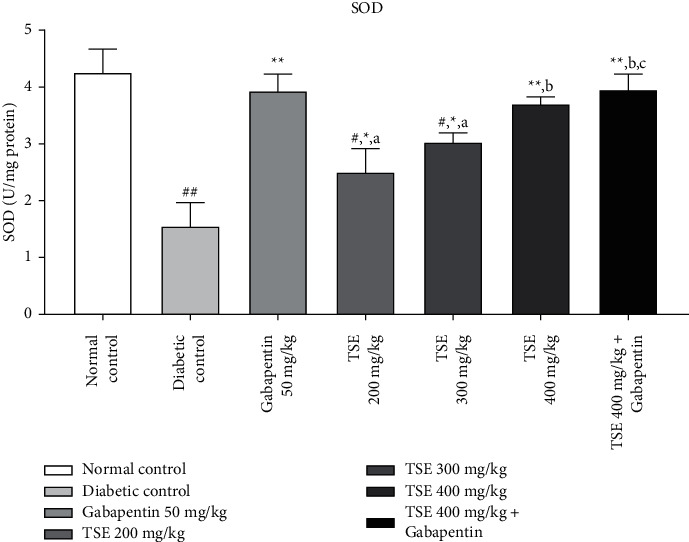
Estimation of SOD levels after completion of the study protocol. Data are expressed as mean ± SD (*n* = 6). Data were statistically analyzed using one-way ANOVA followed by Tukey–Kramer's multiple comparison test at *p* < 0.05, *p* < 0.01, and *p* < 0.001. The values are expressed as ^#^*p* < 0.05 and ^##^*p* < 0.01 compared with the normal control group; ^*∗*^*p* < 0.05 and ^*∗∗*^*p* < 0.01 compared with the diabetic control group; ^*a*^*p* < 0.05 compared with standard treatment, Gabapentin; ^*b*^*p* < 0.05 compared with TSE 200 mg/kg; and ^*c*^*p* < 0.05 compared with TSE 300 mg/kg.

STZ-injected rats depicted a momentous downfall in SOD levels implicating conditions with severe oxidative stress. Administration of *T. stans* extract at 200 mg/kg, 300 mg/kg, and the highest dose, i.e., 400 mg/kg, along with the standard treatment Gabapentin in diabetic rats significantly upregulated the elevation of SOD against the disease control group [36]. The combination produced nonsignificant results vs. Gabapentin-treated group as shown in Figure 7.

#### 3.4.3. Estimation of TBARS Levels

**Figure 8:**
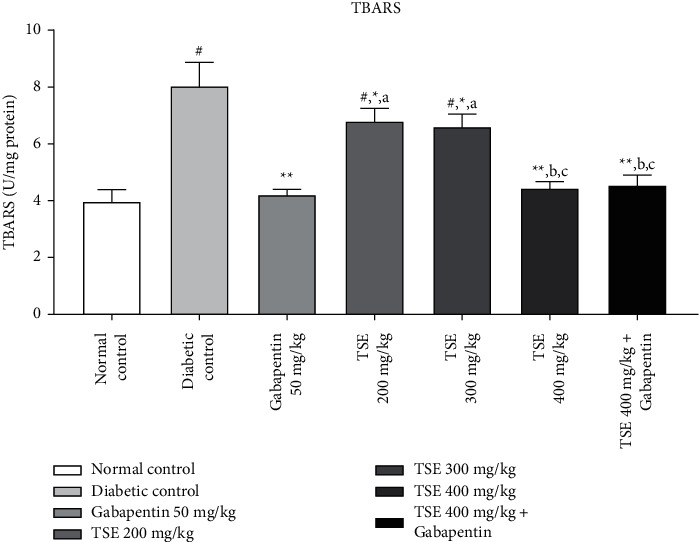
Estimation of TBARS levels after completion of the study protocol. Data are expressed as mean ± SD (*n* = 6). Data were statistically analyzed using one-way ANOVA followed by Tukey–Kramer's multiple comparison test at *p* < 0.05, *p* < 0.01, and *p* < 0.001. The values are expressed as ^#^*p* < 0.05 and ^##^*p* < 0.01 compared with the normal control group; ^*∗*^*p* < 0.05 and ^*∗∗*^*p* < 0.01 compared with the diabetic control group; ^*a*^*p* < 0.05 compared with standard treatment, Gabapentin; ^*b*^*p* < 0.05 compared with TSE 200 mg/kg; and ^*c*^*p* < 0.05 compared with TSE 300 mg/kg.

It has been seen from the literature evidence that administration of STZ leads to the substantial increase in the level of serum MDA, which is considered to be a marker for severe oxidative stress. Administration of *T. stans* extract at 200 mg/kg, 300 mg/kg, and the highest dose, i.e., 400 mg/kg, along with the standard treatment Gabapentin was shown to significantly decrease the level of MDA in the treatment group in comparison to the disease control group [37]. The combination produced statistically nonsignificant results in comparison to Gabapentin as shown in Figure 8.

### 3.5. Determination of Behavioural Parameters

#### 3.5.1. Cold Allodynia Using the Acetone Test

In this experiment, acetone drops (50 *μ*L) were applied gently to the midplantar surface of the hind paw of Wistar rats for all groups. Reaction in the form of paw licking, rubbing, or shaking movement of the hind paw and even food withdrawal was recorded as a means of nociceptive pain response to the cold stimuli. The response time was noted with a digital stopwatch (1 min duration) after the application of acetone. For each reading, both paws were sampled 3 times and the mean was calculated. The results are depicted in Table 1.

#### 3.5.2. Hot Allodynia Using Eddy's Hot Plate

This method involves the estimation of the nociceptive response of methanolic extract of *T. stans*. Each animal from all groups was placed on a hot plate, which was maintained at a constant temperature of 55°C. The reaction in the form of paw licking or jumping was taken with the help of a digital stopwatch and considered a positive response to the hot stimuli, and these responses were considered as a feedback response as thermal hyperalgesia. The animals exceeding the cutoff time were removed from the hot plate till baseline values were achieved. The results are depicted in Table 2.

#### 3.5.3. Grip Strength Test Using Rotarod

This test is used to estimate the muscle strength by gripping the rotarod, and falling-off time is noted. Since this method involves the principle of analyzing muscle strength, muscle relaxation is noted in the animal models of diabetic neuropathy. For this experiment, a fixed speed of 20 to 25 rpm is maintained. The results are depicted in Table 3.

#### 3.5.4. Hyperalgesia Using Tail Flick

This method involves hyperalgesia induced by heat and involves withdrawal of Wistar rat tail, which is considered an endpoint. Following the protocol, any animal which fails to withdraw its tail within 5 seconds is rejected from the experiment. After the administration of the test drug and standard drug, the reaction time was noted after 30 minutes. The results are depicted in Table 4.

## 4. Discussion

From the literature survey, it was concluded that *T. stans* has shown promising results and beneficial effects in the treatment of diabetes and management of associated hyperglycemia [10]. The effects were not only limited to management of glucose levels but also have shown promising results as antioxidants. *T. stans* flowers, its leaves, and the complete plant have been used by various scientists in the management of various metabolic disorders due to their antioxidant activity and radical scavenging activity, which are also associated with decreasing blood glucose levels. These effects are mainly mediated by the presence of various chemical constituents including monoterpenoid alkaloids (specifically tecostanine and tecomanine), phenolics compounds (sinapic acids, o-coumaric, vanillic, caffeic, and chlorogenic), p-sitosterol, triterpenoids (*α*-amyrin, oleanolic, and ursolic acids), and lapachol [10]. The combined effects of these might prove beneficial in the management of various metabolic disorders.

In this study, we hypothesize that *T. stans* leaves could help in the management of diabetes-associated complications of neuropathy. Hence, this study was undertaken to explore the potential of *T. stans* extract in the management of diabetic-associated complications of neuropathy. Also, we evaluated the modulation of glycemic, angiogenic, inflammatory, and oxidative pathways in the pathogenesis of diabetic neuropathy. The methanolic extract of *T. stans* was used based on the literature findings, as most of the active constituents which includes complex indole alkaloids were found to be more soluble in the methanolic extract.

Pathogens act as a trigger to activate phagocytes which ultimately results in the activation of the immune complex leading to the generation of highly toxic reactive oxygen species (RoS), which are responsible for the ingestion of pathogens. It has been seen that NADPH oxidase mediates increased consumption of oxygen, which is required for the production of superoxide radicals and ultimately results in the oxidative burst [38]. From the literature studies and the available data, various oxidants such as nitric oxide radical, hydrogen peroxide, and peroxynitrite radicals are responsible for the induction of oxidative stress in the case of diabetic neuropathy [39]. These agents serve as a trigger for the generation of oxidative stress in various animal models as well. The peroxidation byproducts of lipids are used for the estimation of RoS [40]. Also, the polyunsaturated fatty acids are peroxidized by MDA which acts as a stabilizing agent in the cellular membrane. In conditions of inflammation, the level of MDA is found to be significantly increased. This condition holds true for the animal model of diabetic neuropathy as well.

Wistar rats with STZ-induced diabetes were employed as an experimental model where the study was capped as 28 days for the development of diabetic neuropathy. The study had ten groups with six rats in each. Gabapentin (10 mg/kg) was used as a standard antidiabetic drug in the treatment of diabetic neuropathy. As evident from the literature, Gabapentin exhibits its effect as an antioxidant and anti-inflammatory agent, the combined effect of which resulted in decreased blood glucose levels; however, it may not be sufficient in comparison to the standard care of treatment for diabetes mellitus alone [41].

Methanolic extract of *T. stans* leaves at different concentrations of 200 mg/kg, 300 mg/kg, and 400 mg/kg was employed as a test drug, and the effects were compared with the standard care of treatment with Gabapentin in diabetic neuropathy. For the general parameters, blood glucose was estimated weekly for a period of 4 weeks. Thereafter, animals were sacrificed after the 4th week and the blood was collected via cardiac puncture. Various biomarkers for oxidative stress, angiogenesis, and inflammation were evaluated using ELISA kits.

*T. stans* at various doses (200, 300, and 400 mg/kg) in lieu of its antioxidant effects significantly demolished the levels of MDA and was found to be responsible to safeguard the structural integrity of the cellular membranes. Also, the levels of biomarkers including catalase and SOD which were decreased significantly in disease control groups were embarked with normalization following the treatment of *T. stans* extract. To establish the decreased pain threshold as determined by increased flinching in the formalin test and decreased withdrawal latency in hot plate and tail flick tests, which is a known secondary complication of DN, behavioral parameters which included hot and cold hyperalgesia along with tail flick were analyzed. Similarly, the rotarod test was employed to assess the effect of treatment on motor coordination. Nonsignificant differences were seen for TSE at 300 and 400 mg/kg when compared with the standard treatment. Also, the combination therapy brings similar results in line with the standard treatment of Gabapentin. Thus, it may be concluded based on the findings and available literature that the pharmacological activity possessed by the *T. stans* is mainly attributed to the presence of alkaloids, flavonoids, and glycosides, which act as an active pharmaceutical ingredient for these therapeutic mechanisms showing antioxidant, antiangiogenic and anti-inflammatory properties.

## 5. Conclusion

*T. stans* methanolic extract has provided promising results as an antioxidant, antiangiogenic, and anti-inflammatory mainly at 300 mg/kg and 400 mg/kg doses, an activity in line with the standard drug Gabapentin. Moreover, the methanolic extract of *T. stans* has shown tremendous potential as an immune-modulating agent and was found to significantly affect the levels of inflammatory cytokines which included TNF-*α* as an IL-1*β*.

The herbal drugs appear to prevent oxidative stress-mediated damage by altering the responses of endogenous enzymes of antioxidant, thereby highlighting beneficial effects. The concentrations of thiobarbituric acid reactive substances (TBARS) were lowered on treatment, while the activity of catalase and SOD was increased. The concentrations of angiogenic parameters VEGF and PKC were also lowered in treated animal groups as compared to control groups indicating the beneficial effect of the herbal extract in attenuating angiogenesis mediated diabetes-associated neuropathy. Similarly, promising results were obtained from behavioral parameters, where TSE (300 and 400 mg/kg) produced nonsignificant differences when compared with the standard treatment, depicting its antihyperalgesia and effect of motor coordination.

Therefore, the study evidently signifies the effective role of methanolic extract of *T. stans* in the management of neuropathy by acting as antioxidant, antiangiogenic, and anti-inflammatory agents and thus further research will warrant the ethnomedicinal use of this plant in the management of diabetic neuropathy.

## Figures and Tables

**Table 1:** Effect of *Tecoma stans* extract on cold allodynia.

	Normal Control	Diabetic Control	Gabapentin 50 mg/kg	TSE 200 mg/kg	TSE 300 mg/kg	TSE 400 mg/kg	TSE 400 mg/kg + Gabapentin
0 Week	4.42 ± 0.21	4.21 ± 0.51	4.65 ± 1.22	4.35 ± .051	4.55 ± 0.51	4.75 ± 1.6	4.71 ± 1.8
Week 1	4.45 ± .051	5.98 ± 1.82	5.45 ± 1.12 ^*∗*^	5.95 ± 1.74 ^+^	5.91 ± 1.5 ^+^	5.60 ± 0.40 ^*∗*^^,+^	5.49 ± 1.25 ^*∗*^
Week 2	4.65 ± 1.22	6.89 ± 1.25	5.15 ± 5.62^*∗∗*^	5.62 ± 1.72 ^*∗∗*^^,+^	5.71 ± 0.30 ^*∗∗*^^,++^	5.36 ± 1.87 ^*∗∗*^^,+^	5.16 ± 1.30 ^*∗*^
Week 3	4.63 ± 1.82	7.88 ± 1.92	4.85 ± 6.12^*∗∗*^	5.20 ± 0.41^*∗∗*^^,+^	5.25 ± .1.7 ^*∗∗*^^,+^	5.10 ± 1.67 ^*∗∗*^	5.05 ± 1.32 ^*∗∗*^
Week 4	4.55 ± 0.51	9.78 ± 1.52^##^	4.42 ± 1.52^*∗∗∗*^	4.93 ± 0.51^##,^^*∗∗*^^,++^	4.83 ± 1.12^##,^^*∗∗*^^,+^	4.69 ± 0.14 ^*∗∗*^	4.47 ± 0.68 ^*∗∗*^

Data are expressed as mean ± SD (*n* = 6). Data were statistically analyzed using two-way ANOVA followed by Tukey–Kramer's multiple comparison test at *p* < 0.05, *p* < 0.01, and *p* < 0.001. The values are expressed as ^#^*p* < 0.05 and ^##^*p* < 0.01 compared with the normal control group at the 4th week; ^*∗*^*p* < 0.05, ^*∗∗*^*p* < 0.01, and ^*∗∗∗*^*p* < 0.001 compared with the diabetic control group; ^+^*p* < 0.05, ^++^*p* < 0.01, and^+++^*p* < 0.001 compared with standard treatment Gabapentin.

**Table 2:** Effect of *Tecoma stans* extract on thermal hyperalgesia.

	Normal Control	Diabetic Control	Gabapentin 50 mg/kg	TSE 200 mg/kg	TSE 300 mg/kg	TSE 400 mg/kg	TSE 400 mg/kg + Gabapentin
0 Week	4.21 ± 0.21	4.51 ± 0.51	4.83 ± 1.2	4.85 ± 1.22	4.73 ± 1.82	4.75 ± 1.6	4.81 ± 1.8
Week 1	4.32 ± .051	5.88 ± 1.82	5.42 ± 1.12	5.75 ± 1.74 ^+^	5.85 ± 1.5 ^+^	5.43 ± 0.40	5.34 ± 1.25 ^*∗*^^,aa^
Week 2	4.55 ± 1.22	6.59 ± 1.25	5.05 ± 2.62^*∗∗*^	5.72 ± 1.72^*∗∗*^^,++^	5.69 ± 2.30 ^*∗∗*^^,++^	5.29 ± 1.87 ^*∗∗*^^,+^	5.11 ± 1.30 ^*∗∗*^^,aa^
Week 3	4.53 ± 1.82	7.58 ± 1.92	4.95 ± 3.12^*∗∗*^	5.25 ± 0.41^*∗∗*^^,++^	5.23 ± .1.7 ^*∗∗*^^,+^	5.09 ± 1.67 ^*∗∗*^	5.01 ± 1.32^*∗∗*^^,a^
Week 4	4.35 ± 0.51	9.58 ± 1.52^##^	4.48 ± 1.52 ^*∗∗∗*^	4.92 ± 0.51^##,^^*∗∗*^^,++^	4.88 ± 1.12^##,^^*∗∗*^^,+^	4.75 ± 0.14^#,^^*∗∗*^	4.45 ± 0.68^*∗∗*^^,aa^

Data are expressed as mean ± SD (*n* = 6). Data were statistically analyzed using two-way ANOVA followed by Tukey–Kramer's multiple comparison test at *p* < 0.05, *p* < 0.01, and *p* < 0.001. The values are expressed as ^#^*p* < 0.05 and ^##^*p* < 0.01 compared with the normal control group at the 4th week; ^*∗*^*p* < 0.05, ^*∗∗*^*p* < 0.01, and ^*∗∗∗*^*p* < 0.001 compared with the diabetic control group; ^+^*p* < 0.05, ^++^*p* < 0.01, and ^+++^*p* < 0.001 compared with standard treatment Gabapentin; and ^*a*^*p* < 0.05 and ^*aa*^*p* < 0.01 compared with TSE 200 mg/kg.

**Table 3:** Effect of *Tecoma stans* extract on the grip strength test.

	Normal Control	Diabetic Control	Gabapentin 50 mg/kg	TSE 200 mg/kg	TSE 300 mg/kg	TSE 400 mg/kg	TSE 400 mg/kg + Gabapentin
0 Week	124.21 ± 2.21	124.51 ± 2.51	123.85 ± 3.22	128.85 ± 2.76	126.73 ± 1282	124.75 ± 2.6	127.81 ± 2.8
Week 1	125.32 ± 2.51	69.88 ± 4.82	75.35 ± 1.12^*∗*^	74.75 ± 2.74	73.85 ± 2.52 ^+^	79.55 ± 4.40 ^*∗*^^,+^	74.50 ± 4.25 ^*∗*^
Week 2	123.85 ± 3.22	52.59 ± 2.25	83.05 ± 5.62^*∗∗*^	70.72 ± 4.12 ^*∗∗*^^,+^	79.69 ± 3.30^*∗∗*^^,++^	85.29 ± 4.87 ^*∗∗*^^,+,a^	82.11 ± 5.30 ^*∗*^^,aa^
Week 3	129.73 ± 3.82	35.58 ± 4.92	99.95 ± 6.12^*∗∗*^	75.25 ± 3.41 ^*∗∗*^^,++^	80.15 ± .2.71 ^*∗∗*^^,++^	89.09 ± 2.67 ^*∗∗*^^,aa^	98.01 ± 4.32 ^*∗∗*^^,aa^
Week 4	128.25 ± 3.51	23.58 ± 3.52^##^	114.38 ± 2.52^*∗∗∗*^	84.93 ± 4.51^##,^^*∗∗*^^,+++^	94.88 ± 3.12^##,^^*∗∗*^^,+^	104.75 ± 3.14^#,^^*∗∗∗*^^,aa^	116.45 ± 3.68 ^*∗∗*^^,aaa^

Data are expressed as mean ± SD (*n* = 6). Data were statistically analyzed using two-way ANOVA followed by Tukey–Kramer's multiple comparison test at *p* < 0.05, *p* < 0.01, and *p* < 0.001. The values are expressed as ^#^*p* < 0.05 and ^##^*p* < 0.01 compared with the normal control group at 4th week; ^*∗*^*p* < 0.05, ^*∗∗*^*p* < 0.01, and ^*∗∗∗*^*p* < 0.001 compared with the diabetic control group; ^+^*p* < 0.05, ^++^*p* < 0.01, and ^+++^*p* < 0.001 compared with standard treatment Gabapentin; ^*a*^*p* < 0.05 and ^*aa*^*p* < 0.01 compared with TSE 200 mg/kg.

**Table 4:** Effect of *Tecoma stans* extract on thermal hyperalgesia using tail flick.

	Normal Control	Diabetic Control	Gabapentin 50 mg/kg	TSE 200 mg/kg	TSE 300 mg/kg	TSE 400 mg/kg	TSE 400 mg/kg + Gabapentin
0 Week	7.21 ± 0.21	7.51 ± 2.51	7.34 ± 1.05	7.40 ± 1.08	7.61 ± 1.21	7.32 ± 1.51	7.52 ± 1.51
Week 1	7.32 ± 1.51	6.48 ± 4.82	6.35 ± 1.12	6.25 ± 2.74	6.30 ± 2.5	6.32 ± 4.40	6.50 ± 4.25
Week 2	6.85 ± 1.22	5.59 ± 2.25	6.55 ± 1.62^*∗*^	6.36 ± 4.12^*∗∗*^^,+^	6.40 ± 3.30 ^*∗∗*^^,+^	6.59 ± 4.87 ^*∗∗*^^,a^	6.57 ± 5.30 ^*∗∗*^^,aa^
Week 3	6.73 ± 0.82	5.08 ± 4.92	6.95 ± 1.12^*∗∗*^	6.59 ± 3.41^*∗∗*^^,+^	6.64 ± .2.7 ^*∗∗*^^,+^	6.89 ± 2.67 ^*∗∗*^^,a^	6.91 ± 4.32^*∗∗*^^,aa^
Week 4	7.25 ± 1.51	4.58 ± 3.52^##^	7.09 ± 2.52^*∗∗∗*^	6.73 ± 4.51^##,^^*∗∗∗*^	6.88 ± 3.12^#,^^*∗∗*^^,+^	6.95 ± 3.14 ^*∗∗∗*^^,a^	7.03 ± 3.68^*∗∗*^^,aa^

Data are expressed as mean ± SD (*n* = 6). Data were statistically analyzed using two-way ANOVA followed by Tukey–Kramer's multiple comparison test at *p* < 0.05, *p* < 0.01, and *p* < 0.001. The values are expressed as ^#^*p* < 0.05 and ^##^*p* < 0.01 compared with the normal control group at the 4th week; ^*∗*^*p* < 0.05, ^*∗∗*^*p* < 0.01, and ^*∗∗∗*^*p* < 0.001 compared with the diabetic control group; ^+^*p* < 0.05, ^++^*p* < 0.01, and ^+++^*p* < 0.001 compared with standard treatment Gabapentin; and ^*a*^*p* < 0.05 and ^*aa*^*p* < 0.01 compared with TSE 200 mg/kg.

## Data Availability

The data used or analyzed during the study are available from the corresponding author.
